# A rat in the sewer: How mental imagery interacts with object recognition

**DOI:** 10.1371/journal.pone.0194227

**Published:** 2018-03-28

**Authors:** Harun Karimpur, Kai Hamburger

**Affiliations:** 1 Experimental Psychology, Justus Liebig University, Giessen, Germany; 2 Experimental Psychology and Cognitive Science, Justus Liebig University, Giessen, Germany; Public Library of Science, UNITED KINGDOM

## Abstract

The role of mental imagery has been puzzling researchers for more than two millennia. Both positive and negative effects of mental imagery on information processing have been discussed. The aim of this work was to examine how mental imagery affects object recognition and associative learning. Based on different perceptual and cognitive accounts we tested our *imagery-induced interaction hypothesis* in a series of two experiments. According to that, mental imagery could lead to (1) a superior performance in object recognition and associative learning if these objects are imagery-congruent (semantically) and to (2) an inferior performance if these objects are imagery-incongruent. In the first experiment, we used a static environment and tested associative learning. In the second experiment, subjects encoded object information in a dynamic environment by means of a virtual sewer system. Our results demonstrate that subjects who received a role adoption task (by means of guided mental imagery) performed better when imagery-congruent objects were used and worse when imagery-incongruent objects were used. We finally discuss our findings also with respect to alternative accounts and plead for a multi-methodological approach for future research in order to solve this issue.

## Introduction

“You are what you eat”–this well-known proverb (directed to our dietary habits), in a more abstract sense, stands for the idea that our actions influence our body and our personality. At the same time, we see that our thoughts also have an influence on us such as it is the case with guided imagery. It is a guided form of mental imagery that can, for example, be found in clinical settings. So, what if you become as good as you are told you are? What if imagining of being a rat in the sewer, improves the way you perceive and recognize conceptually related objects? Focusing on spatial cognition, this work examines the role of mental imagery and its influence on the recognition of salient objects along a route, so-called *landmarks*. By systematically manipulating landmark types and role adoption tasks, we will show that the effect of mental imagery can influence subsequent encoding and recognition processes of landmarks. Whether this effect facilitates or impedes these cognitive abilities depends on the semantic congruence between landmark and adopted role, as we shall later see. Our findings suggest that we need to be cautious and that more research is needed in order to understand when detrimental effects of mental imagery occur.

In the following, we will first present our theoretical foundation before we derive our hypothesis from that. For readability purposes, we will divide them into three subsections: (1) mental imagery & perception, (2) mental imagery & semantic salience and (3) mental imagery & memory.

### Mental imagery & perception

Mental imagery has been a controversial topic for more than two-thousand years. Long before it was in the focus of modern psychologists, we find thoughts on this topic in the writings of early philosophers. In *Theaetetus*, Plato developed the *wax tablet metaphor* which described our memories as the imprinted information on a wax tablet [1]. His most famous student, Aristotle, extended this idea and strongly linked memory with sense-perception and the act of recollecting with the search for an image [2]. Many centuries later, in 1690, during the Baroque period, John Locke described our ideas as pictures in our minds that vanish if not refreshed [3]. All these different ideas set the foundation of an ongoing debate between psychologists in modern times.

The imagery debate found another peak in the 1970s with Stephen Kosslyn and Zenon Pylyshyn. Kosslyn et al. [4], for example, took the view that empirical evidence, such as classic findings of Shepard et al. [5] on mental rotation, corroborates their view on mental imagery and its function to depict. Pylyshyn’s propositional account did not question the experience of mental images per se, it questioned mental imagery as a form of memory representation. According to this view, the experience of mental imagery is represented in the same form as language [6]. He further argues that a sensory or pictorial view cannot account for abstract knowledge while a propositional account could, and that the images we experience are epiphenomena.

This study does not aim to take sides. However, if mental imagery and perception share the same underlying processes, the implications of Kosslyn’s view about quasi-pictorial representations would be that guided imagery could leave imprints in what he calls the *visual buffer*. This in turn could facilitate the encoding and recognition of objects that are related to what has been imagined. Additionally, an occupied buffer could hinder the effective processing of unrelated visual information. While this *exact* process lacks of empirical substance, we see others who state related ideas. For instance, Rebotier et al. [7] describe such an effect in the beginning of their paper:

[…] *suppose that you are visiting the Louvre and get a glimpse of a painting through a closing door*. *Expecting to see the Mona Lisa in that particular location*, *you would be visualizing Da Vinci’s masterpiece*. *If the Mona Lisa is there*, *that ongoing visualization should help you see it*. *On the other hand*, *had the museum swapped the Mona Lisa with an El Greco landscape*, *you would not identify this landscape because your visual pathways would be biased in favor of the Mona Lisa*. (p. 343)

**Fig 1:**
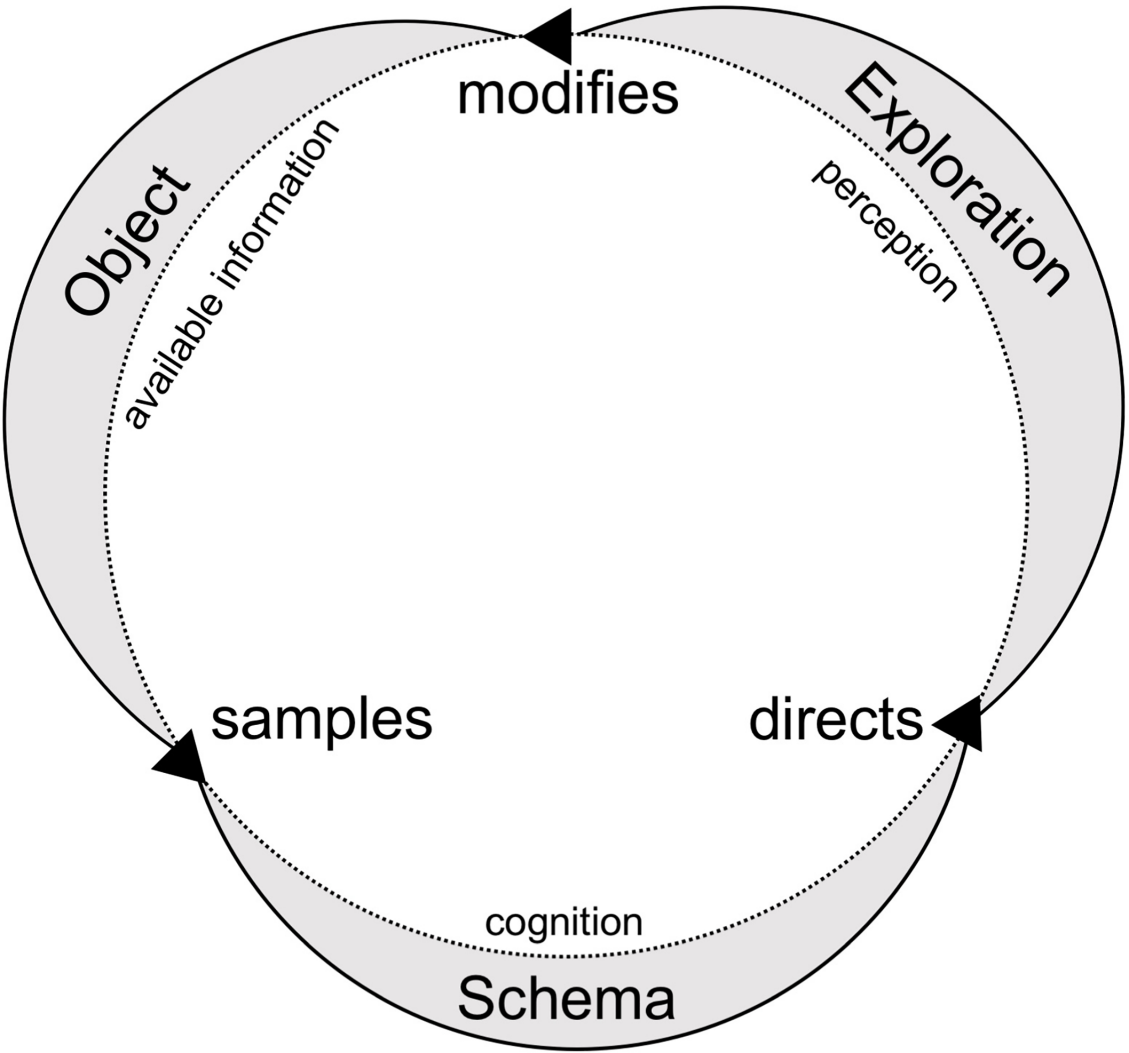
The perceptual cycle model. Adapted from Neisser, 1976.

This very idea can also be found in Neisser’s theoretical framework who, however, opposed both Kosslyn and Pylyshyn with his alternative approach [8]. He insisted that the act of imagining is not perceiving but that we derive these images from perceptual activity. According to his hypothesis, mental imagery leads to a readiness to perceive certain information rather than other (anticipatory phase), and are described as detached schemata from the perceptual cycle. The perceptual cycle (Fig 1) is a model that describes the relationship between schemata, exploration and objects. Active schemata are anticipations of certain kinds of information. These anticipatory schemata in turn direct how we explore our environment and perceive objects. What we explore (i.e., the obtained object information) then modifies the original schema. Today, the perceptual cycle model, that addresses both bottom-up and top-down effects, is widely accepted and could even be validated in the context of aeronautics [9].

Now it is time to mention the other side: mental imagery can also hinder subsequent object recognition. Neisser pointed to studies that reported facilitated identification of capital letters after imagining them and hindered identification when these letters were presented in lower case [10]. Peterson et al. [11] used a visual detection task and found a similar pattern, i.e., cue-compatible mental imagery enhanced detection, cue-incompatible mental imagery interfered. This interaction was also found in studies that used both behavioral and functional magnetic resonance imaging (fMRI) methods. For example, reduced reaction times (priming on a behavioral level) and reduced activity in inferior prefrontal brain regions (priming on a neural level) was associated with lower levels of subsequent explicit memory. The authors concluded that priming can indeed hinder new episodic encoding [12].

The problem with the aforementioned findings is that all of them were conducted with a small spatio-temporal window in mind. However, it was Neisser who already emphasized (1) the importance of movement and space during the exploration phase of the perceptual cycle model and (2) the abstractness of schemata [8]. Therefore, the question that arises is: does that interaction also hold on a greater spatio-temporal dimension and on a broader conceptual level? We decided to take the above findings as one foundation of our study. Based on that, we expect that prior mental imagery will affect subsequent object encoding and recognition depending on the semantic congruence between objects and the content of the mental representation. In order to test this hypothesis, we will use methods from the field of spatial cognition. In fact, semantics and semantic congruence are important factors that find their place also in theoretical frameworks of landmark-based wayfinding, as we will see in the following section.

### Mental imagery & semantic salience

Spatial orientation and wayfinding has been well studied [13, 14]. In the past years, a lot of research has been conducted in regards to spatial cognition in animals as well as in humans. For example, spatial orientation, navigation and its strategies, the role of memory, and neural substrates of spatial cognition were examined [15].

In our current study, we want to extend the line of research, which aims to understand how mental imagery affects landmark information processing. Landmarks, in general, are defined as objects (e.g., a red phone booth on a corner), which stand out from the environment and can therefore act as reference points [16]. Of course, wayfinding can be conducted without landmarks. However, the recognition of landmarks remains and essential cognitive process that allows us to associate the landmark with a certain direction [17].

In principle, the extent to which a landmark stands out from its environment is called landmark salience. However, it should not only be seen as the property of certain spatial features. Based on a trilateral relation between the observer’s point of view, the feature itself and the surrounding environment, Caduff et al. [18] described landmark salience as a unique property of the aforementioned on a cognitive level as well as on a physical level.

In regards to landmark salience, a distinction between visual, semantic and structural salience is made [19, 20]. First, the term *visual salience* describes visual object properties (e.g., color, shape, size, etc.). Accordingly, the salience of a bright shining big white sphere in a dark environment would be classified as high. Second, the term *structural salience* stands for navigation-related features (e.g., location of a landmark at an intersection). For example, a statue in the center of a roundabout or an object in front of an intersection in direction of turn would be considered as having a high structural salience [21]. Third, the term *semantic salience* describes knowledge-related properties (e.g., fame, prominence, function, etc.). A landmark with a high semantic salience could be the Eiffel Tower in Paris (at least on a semantic level only for those who know the Eiffel Tower).

Concerning the semantic salience, which Caduff et al. [18] also called cognitive salience, it is important to note that it also refers to idiosyncratic relevance and thus represents a very individual form of landmark salience (e.g., my favorite bakery, the house a former girlfriend lived in). For our study, this is of great importance, especially the notion of idiosyncratic relevance. For example, one might ask: “Is the Statue of Liberty the same to you as it is to your friend?”–of course not. This individual level needs consideration when it comes to semantic landmark salience. Its conceptual fuzziness is a reason why researchers in the field of spatial cognition might hesitate to examine this topic more thoroughly. However, for our study, it is the very idiosyncrasy which allows us to connect the role of landmarks with mental imagery. In this case, mental imagery could be used to adopt a role, which could lead to a shift in the idiosyncratic relevance. That in turn could additionally cause that semantically related objects are more salient than others are. If we can increase the salience of a landmark, it should also affect the way information is being processed and memorized. For example, we could show that certain affective states can have such an effect [22]. This is of particular interest because of the function of a landmark in regards to associative learning (landmark information connected to directions). Therefore, in the next section, we will present findings with respect to learning and memory.

### Mental imagery & memory

Mental imagery is very common in the context of clinical psychology (e.g., Guided Imagery in exposure therapy) and sport psychology (e.g., visually imagining complex actions for practice). In contrast, little is known about the effect of such imagination tasks (from here on: *role adoption tasks*) in other fields of psychological research. Before researchers began to investigate the influence of mental imagery on memory, they started to focus on the role of perspective. For example, Pichert et al. [23] could show that learning and recalling information depends on an idea’s significance, and more importantly, on the perspective from which a given story is being told. However, that is not fully comparable to imagination tasks from which we know that they are guided and aim to let people identify themselves with a situation and all of its consequences (e.g., empathy, emotions).

One of the first studies using mental imagery to influence memory was from Sahakyan et al. [24]. Based on findings in regards to context-dependent memory effects [25] and the *Theory of Intentional Forgetting* [26], the authors claimed that forgetting can be a result of changes in mental context. This change in internal context was achieved by mental imagery. First, subjects had to learn a list of items. Some of them were then asked to mentally walk through their parent’s house before learning a second list of items. The authors found support for the so-called *context change hypothesis of directed forgetting* and showed that a change of the mental context in the absence of a forget instruction leads to results comparable to directed forgetting. However, there is more to the effect of mental imagery on memory. Aslan et al. [27] could show that imagination tasks can impair recall of previously encoded items while improving recall of subsequently encoded items. These findings suggest that imagination tasks can have a positive as well as a negative effect on memory performance. Since memory also plays a crucial role when it comes to navigation in our everyday lives, it is important to understand in which way such tasks affect our memory in this domain. To the best of our knowledge, no work trying to examine the role of mental imagery in the context of landmark recognition has been published yet.

### Aim of the study

The three previous sections demonstrated that mental imagery has the potential to affect information processing positively as well as negatively. The first subsection described this on different perceptual accounts. The second subsection demonstrated that the perception of specific objects (landmarks) are of particular interest because of their semantic salience. The third subsection demonstrated that mental imagery could also facilitate or hinder the retrieval of such information.

We therefore postulate our *Imagery-Induced Interaction Hypothesis* (3IH). The 3IH relates to the effect of mental imagery on information processing. To be more specific, it states that mentally imagining a concept (e.g., by the means of a role adoption task) will facilitate information processing of semantically related objects and impede it in case of semantically irrelevant objects. The term concept stands for an idea that, if applied, significantly affects the idiosyncratic relevance of an object or group of objects. Consequently, we would expect that the idea of being a rat affects the idiosyncratic relevance of objects that are semantically related (e.g., rat poison). We expect the effects on information processing to be reflected in both correct responses and response times.

To test this, we conducted a series of two experiments. In Experiment 1, we examined associative learning in a static context. In Experiment 2, subjects were guided through a virtual environment and were subsequently tested for correct landmark recognition.

## Experiment 1

In Experiment 1, we tested the 3IH by using a static environment. In the experimental group, subjects conducted a role adoption task by means of mental imagery prior to learning stimuli (landmarks) and associated directions. In the control group, subjects did not receive a role adoption task. In the role adoption task, subjects were asked to imagine themselves being a rat living in the sewer (rat group). Both the control and the rat group learned either landmarks that were semantically related to the role adoption task of the experimental group or landmarks that were not so. If the 3IH holds for associative learning, we would expect the rat-group to perform superior in linking imagery-congruent landmarks with directions and perform inferior in case of imagery-incongruent landmarks.

### Methods

#### Subjects

Subjects were 80 students from the Justus Liebig University Giessen, who were recruited via circular mail within the university. Four subjects were excluded after reporting difficulties with the instruction. The mean age of the remaining 76 subjects (62 females, 14 males) was 21.66 years (*SD =* 3.19, ranging from 18–31 years). Previous studies in landmark recognition research already showed that there are no gender effects [e.g., 28]. Subjects provided informed written consent and reported normal or corrected-to-normal visual acuity. All subjects received course credit for participation. The experiment was conducted in accordance with the Declaration of Helsinki and experimental protocols were approved by the local ethics board of the Justus Liebig University Giessen (2014–0017).

#### Material

The role adoption task was presented in German and was conducted by using guided imagery (see S1 Text). First we wrote the exact instructions (hence “guided” imagery) and then audio-recorded these instructions in order to ensure that each subject receives the same words with the same intonation. It consisted of visual information (e.g., “You look down […] and see your tiny paws […]”) and conceptual information about the rat (e.g., “You know these tunnels. They are your home.”).

The landmarks (see S1 Fig) were either imagery-incongruent (pictures of animals) or imagery-congruent (e.g., valves, tunnel entries, etc.). In order to ensure that this was the case, we conducted a preliminary experiment with 80 pictures and let subjects rate these pictures on a 9-point Likert scale (“Imagine you were a rat living in the sewer. To what extent would this picture be relevant to you?”). It is important to note that we did not control whether the content of the images were factually appropriate. That means that a sign reading “caution, rat poison!” could only be relevant for a human being imagining being a rat.

We took the 30 highest rated pictures and randomly chose 15 of them as target stimuli and the other half as distractors. The 30 imagery-incongruent pictures were also randomly divided in 15 target and 15 distractor stimuli. The scene on which these landmarks were presented was depicting an intersection in a sewer.

The experiment was programmed and conducted in MATLAB® Release 2016a (The MathWorks, Inc., Natick, MA) in connection with the Psychophysics Toolbox extensions [29–31].

#### Procedure

The factor *role adoption task* consisted of two levels (*control* and *rat*). The control group did not receive a role adoption task while the rat group did. The factor *landmark type* also consisted of two levels (*imagery-congruent* and *imagery-incongruent*). Hence, subjects were randomly assigned to one of the four conditions of our 2x2 factorial design (between-subjects).

Subjects were seated about 80cm away from a Dell E228WFP 22-inch Widescreen LCD Monitor (1680x1050 pixels screen resolution). Then, subjects of the control group simply waited for a certain amount of time (1min 46s). In case of the role adoption task, subjects wore headphones and began the guided imagery phase (1min 46s). In order to conduct a manipulation check, each subject who received such a treatment was then asked “Could you follow the instructions and visually imagine what you were told to?”.

**Fig 2:**
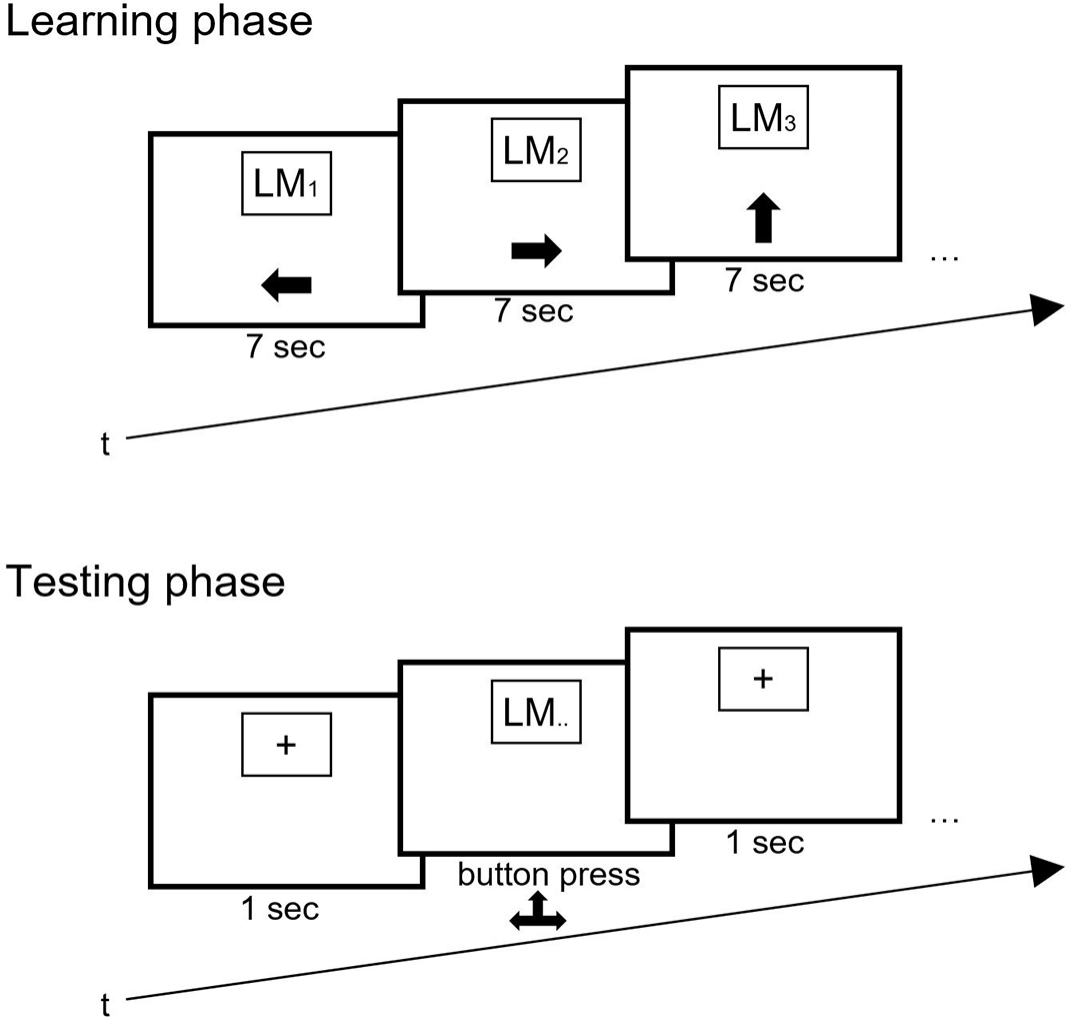
Learning (top) and testing phase (bottom) of Experiment 1. Landmarks (LM) were presented in combination with an arrow pointing either left, straight or right. During testing, landmarks were presented as a cue and subjects indicated the correct direction by pressing the corresponding arrow key.

After that, for each condition, the instructions were presented on the screen and subjects had to confirm that they understood the instructions by pressing a button. The main experiment consisted of two elements: the learning phase and testing phase (Fig 2).

First, the learning phase started. The background depicted an intersection in a sewer system. During each encoding trial, a landmark was presented at the top center of the screen in combination with an arrow (left, straight or right) that was presented below the landmark. Both, landmarks and arrows were presented randomly thus in random combination. The subjects were instructed to learn the landmark-direction combinations. For each encoding trial, subjects had seven seconds. This duration was chosen because it resembled the time that subjects had to encode in such an environment of another experiment between two decision points [32]. The next trial started after an inter-trial-interval of one second. The encoding phase ended after the presentation of all 15 landmark-direction combinations.

Subsequently after the encoding phase, subjects started with the recognition phase. In this phase, we added 15 distractor landmarks to the 15 landmarks that were presented before. Again, during each recognition trial, a landmark was randomly presented at the top center of the screen right after a fixation cross. This time, however, subjects had to (1) indicate the correct direction by pressing the corresponding arrow keys and (2) reject distractor landmarks by pressing the space key. For each trial, subjects were given five seconds before a trial time-out indicated “too slow”. We chose five seconds to allow comparability to prior studies and to limit the possibility of rehearsal. The experiment finished after 30 trials of the recognition phase.

For each trial we measured two dependent variables. First, we measured if a decision was correct. We defined a correct decision as correct landmark-direction combination and correct rejection of distractors. Second, we measured response times for each trial. Responses slower than five seconds were counted as errors.

### Results

**Fig 3:**
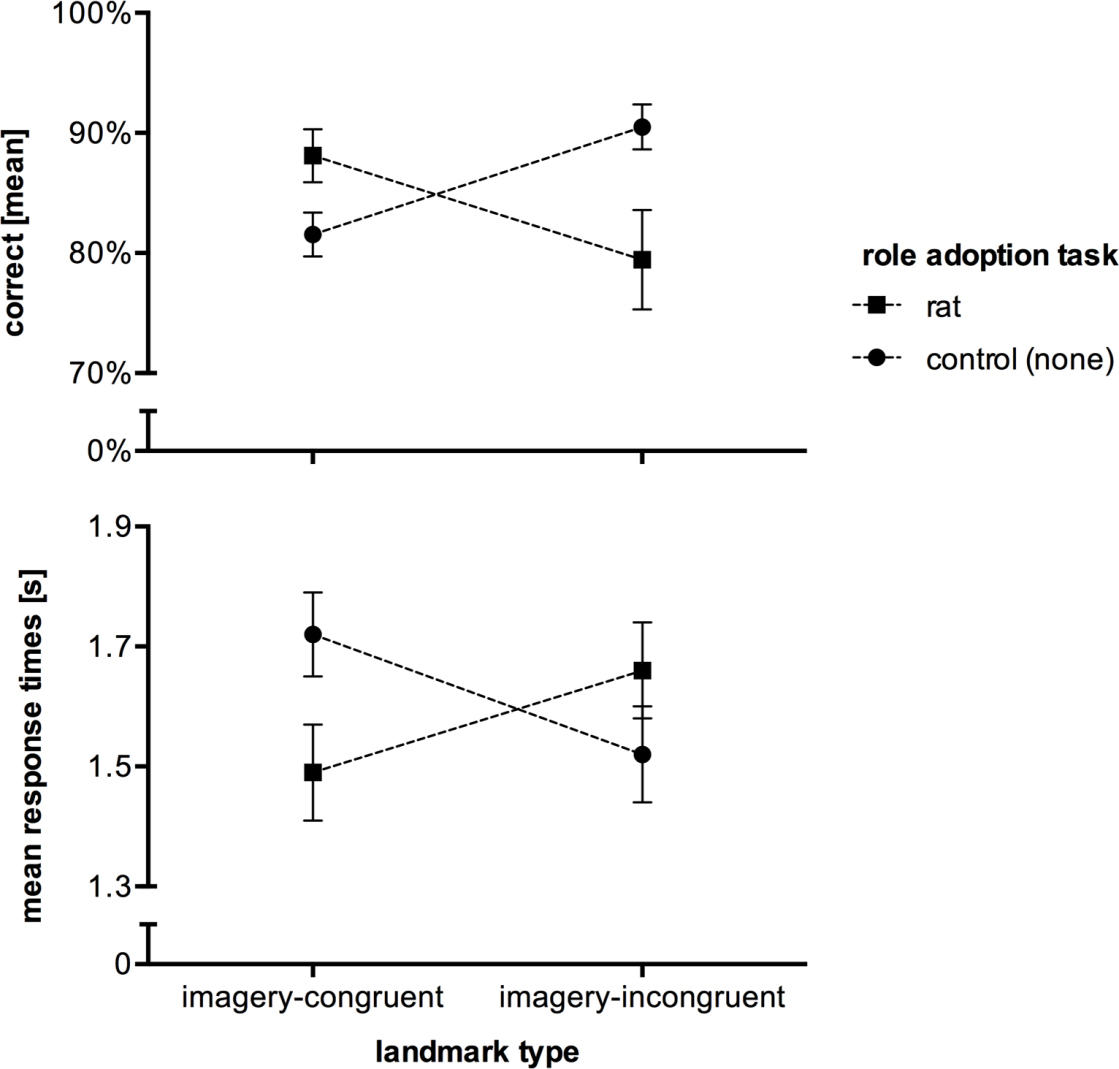
Mean correct responses (top) and response times (bottom) of Experiment 1. Performances of all four *landmark type* x *role adoption task* conditions are depicted. The error bars represent the *SEM*.

All subjects who received a role adoption task confirmed that they could visually imagine what they were told to. Fig 3 depicts the results of our first experiment. The results of our two by two analysis of variance [(control vs rat role adoption task) x (imagery congruent vs incongruent landmarks)] were as follows. In terms of correct decisions, there was neither a significant main effect of landmark type (congruent: *M* = 84.64%, *SEM* = 1.50; incongruent: *M* = 84.70%, *SEM* = 2.48) nor a significant main effect of role adoption task (with: *M* = 83.32%, *SEM*; without: *M* = 86.02%, *SEM* = 1.49; all *F* < 1). However, our results demonstrate a significant interaction between role adoption task and landmark type, *F*(1, 72) = 9.784, *p* = .003, $\eta_{p}^{2}$ = .12. A post-hoc analysis reveals that subjects performed significantly better with a role adoption task in combination with imagery-congruent landmarks compared to imagery-incongruent landmarks (88% vs. 79%, *p* = .033). It further shows that imagery-incongruent landmarks in combination with a role adoption task led to fewer correct decisions compared to without such task (79% vs. 91%, *p* = .006).

In terms of response times, there was a similar pattern (Fig 3; bottom). We did not find a significant main effect of landmark type (congruent: *M* = 1.61 s, *SEM* = 0.06; incongruent: *M* = 1.59 s, *SEM* = 0.06) or role adoption task (with: *M* = 1.58 s, *SEM* = 0.06; without: *M* = 1.62 s, *SEM* = 0.05; all *F* < 1). Again, our results demonstrate a significant interaction between role adoption task and landmark type, *F*(1, 72) = 5.871, *p* = .018, $\eta_{p}^{2}$ = .08. Post-hoc analysis reveals that subjects with a role adoption task were significantly faster compared to subjects without such task when congruent landmarks were used (1.49 s vs. 1.72 s, *p* = .038).

### Discussion

Our first experiment related to the processing of spatial information to the extent that we investigated a key component in landmark-based wayfinding: associative learning of landmarks and directions. It aimed to investigate the 3IH by using a static environment (i.e., images of landmarks on intersections).

Indeed, our results supported our hypothesis for both correct decisions and response times. Subjects in the experimental group used guided mental imagery, i.e., they were told to imagine being a rat living in the sewer. These subjects were better in encoding and retrieving semantically *congruent* landmark information and their corresponding directions compared to subjects who did not receive such a treatment. At the same time, these subjects were worse in encoding and retrieving semantically *incongruent* landmark information and their corresponding directions compared to subjects who did not receive such a treatment.

This pattern was expected according to the 3IH. We see that mental imagery can both facilitate and impede information processing. Such an effect can be explained on a perceptual level by Neisser’s perceptual cycle model. The role adoption task could have led to changes in schemata. These schemata in turn could have facilitated the detection of schemata-congruent stimuli and could have hindered the detection of schemata-incongruent information during the exploration phase. If that was indeed the case, then testing our hypothesis in a more dynamic environment for encoding should also lead to a similar pattern of results, especially if we consider the importance of movement and space for the perceptual cycle model.

Earlier we emphasized the role of semantics and that this could have an influence in regards to landmark recognition. The problem with that is that we did not control for differences in conceptual knowledge about rats. Therefore, in the next experiment, we decided to address the problem of a static environment and the problem of pre-existing knowledge for landmark-recognition.

## Experiment 2

In Experiment 2, our aim was to investigate the Imagery-Induced Interaction Hypothesis (3IH) on landmark recognition in a dynamic environment. Compared to Experiment 1, two major changes were made. First, the encoding phase was now conducted in a virtual environment. Subjects learned landmarks along a path in a virtual sewer system before the pure recognition of landmarks was tested.

Second, next to the control group and the first experimental group (rat) we added a second experimental group. This group also conducted a role adoption task. Instead of imagining being a rat, these subjects were asked to imagine being a fictitious character. We introduced this group in order to control for pre-existing knowledge. For example, in Experiment 1, subjects were told to imagine being a rat with grey fur. This could have led to conflicts with pre-existing knowledge about a rat’s fur. Therefore, by not mentioning the rat explicitly, we hoped to reduce potentially confounding elements of the role adoption task.

### Method

#### Subjects

The sample consisted of 72 students from the Justus Liebig University Giessen (61 females, 11 males), who were also recruited via circular mail. The mean age was 22.72 years (*SD* = 3.19, ranging from 18 to 33 years). All subjects reported normal or corrected-to-normal visual acuity, provided informed written consent and received course credit for participation. Subjects who reported epilepsy (or epilepsy in close relatives) or colorblindness were excluded from the study in advance. This experiment was also done in accordance with the ‘*Declaration of Helsinki*’ and experimental protocols were approved by the local ethics committee (2014–0017).

#### Material

This time, the experimental conditions consisted of two groups with different role adoption tasks. The first one was the same as in Experiment 1 (imagining being a rat). In the second group, subjects were asked to imagine themselves being a fictitious character that we named “squobble”. The wording was exactly the same as it was the case with the first role adoption task. The only difference was that the fur was now described as “pink” and the sentence “You are a rat” was replaced with “You are a squobble”.

**Fig 4:**
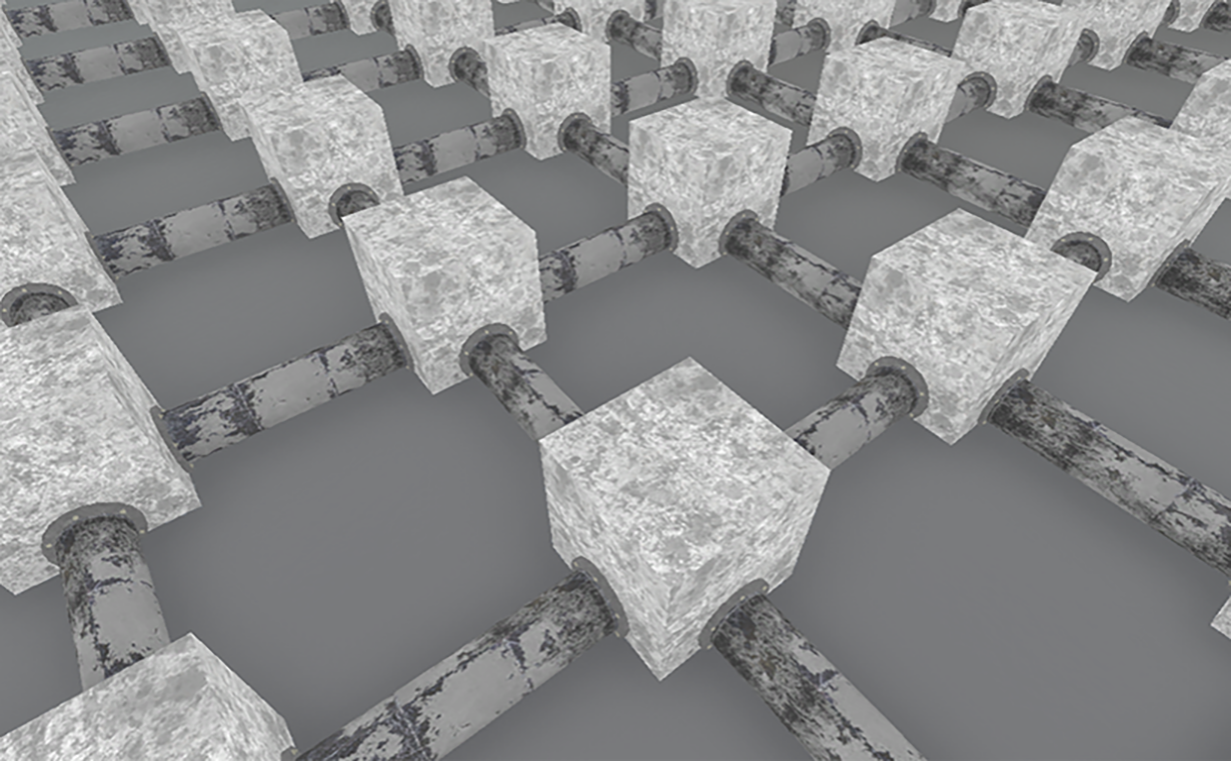
External view of the sewer system that was used in Experiment 2.

The experiment was conducted on a Pentium i7 3.6 GHz computer (16 GB RAM, running Windows 10) using a virtual environment which was programmed with Unreal® Engine 4. The virtual maze (sewer system) with a grid structure was also used in other studies that investigated landmark-based wayfinding [32]. The sewer system was modeled in Autodesk® Maya® 2016. Each sewer segment connected two junctions that had a length of 650 cm. The junctions, that connected the sewers, were of cubic shape with a length of 375 cm (Fig 4).

Subjects wore a head-mounted-display (Oculus Rift™ Developmental Kit 2, Oculus VR, LCC.), which included positional head tracking functionality and a Full HD OLED (Organic Light Emitting Diode) display from which a resolution of 960x1080 pixels was technically visible to each eye. The eye-height was set to 170 cm.

The landmark stimuli were the same as in Experiment 1. They were placed in the middle of each decision point (junction) at a height of 220 cm. For balancing reasons, three routes were developed. By doing so, we could ensure that each landmark was associated with all of the three possible directions on a decision point and that each landmark was presented on each third of the experiment. For example, one stimulus was placed on route one on a right turn and was presented within the first five decision points. On route two, it was placed on a straight and was presented within the last five decision points. Consequently, on route three, it was placed on a right turn and was presented within the central five decision points. All of the 30 landmark stimuli (15 imagery-incongruent landmarks, 15 imagery-congruent landmarks) were placed using this strategy in order to control for directional as well as position biases.

Again, for conducting the recognition task, MATLAB® and the Psychophysics Toolbox extension was used.

#### Procedure

Same as in Experiment 1, this experiment was based on a two factorial between-subjects design. This time, the factor *role adoption task* consisted of three levels (*control*, *rat* and *squobble*) while the factor *landmark type* consisted of two levels (*imagery-congruent* and *imagery-incongruent*) resulting in six conditions. All subjects were randomly assigned to one of these six conditions whereby each individual went through two stages: learning and testing.

**Fig 5:**
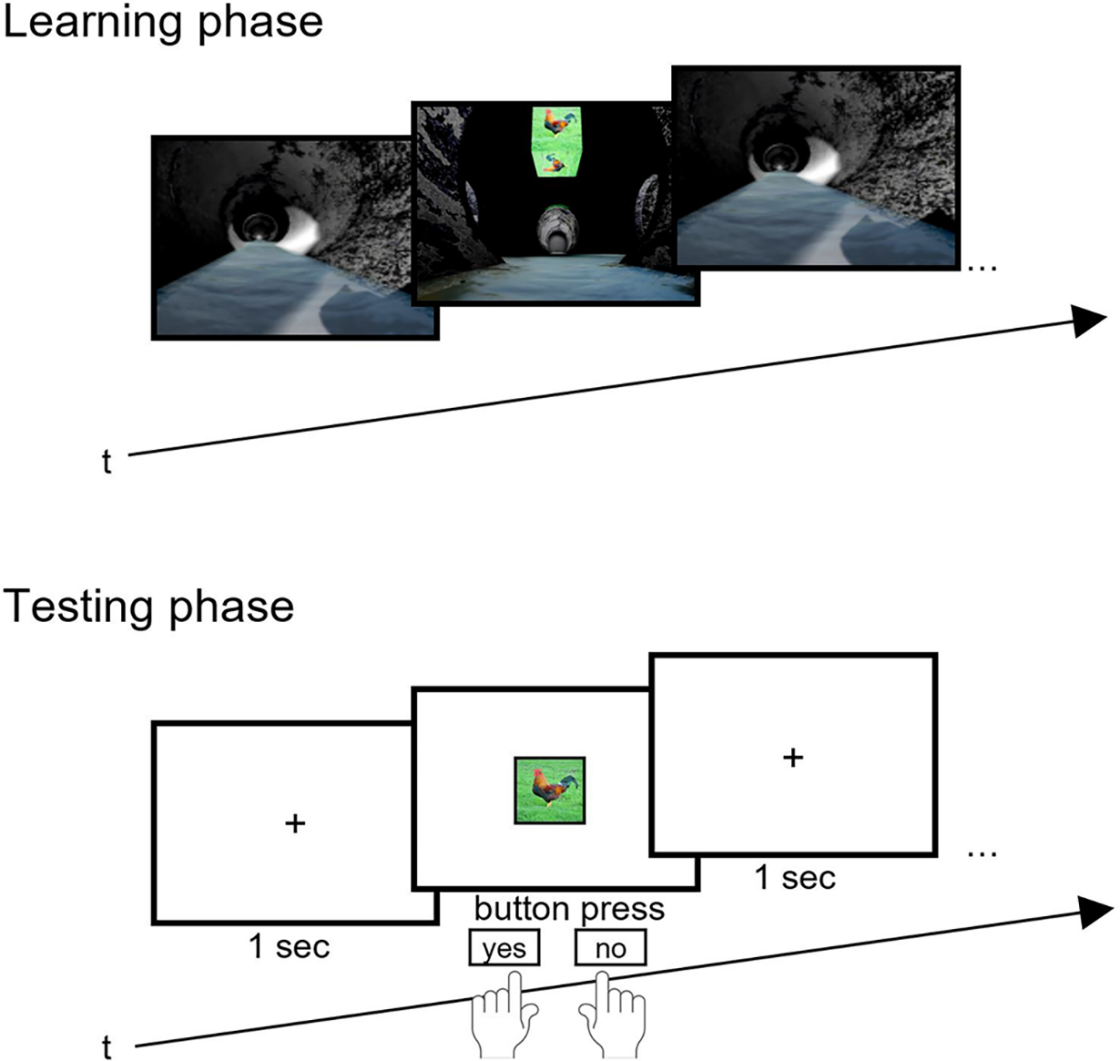
Learning and testing procedures of Experiment 2 with an exemplary landmark (rooster; user *nessmoon* at Morguefile.com). *Top*: subjects were guided through a virtual environment and were instructed to learn a route. Landmarks were placed at each intersection. *Bottom*: Subjects had to indicate whether they had seen the landmark in the environment or not.

First, subjects of the control group waited for a certain amount of time (1min 46s) and subjects in the experimental conditions conducted the role adoption task (see Procedure section of Experiment 1). The experimental task (Fig 5) began with a passive walk through the sewer system, which subjects had to learn (encoding phase). During this phase subjects were able to freely look around (free head movement). This phase was finished after passing 15 landmarks.

This was followed by a recognition task. A total of 30 landmarks (consisting of 15 target stimuli and 15 distractors) were shown in a random order after a fixation cross and subjects were instructed to indicate as fast and accurate as possible whether they had seen them before in the maze or not by pressing a button for “yes” or “no”.

Dependent variables were response accuracy (correct recognition) as well as decision times in the recognition phase.

### Results

All trials were counted as valid and thus no exclusion was necessary. The means of correct decisions were calculated for each condition. They were then compared by using a 2 x 3 [landmark type (imagery-congruent, imagery-incongruent) x role adoption task (control, rat, squobble)] ANOVA.

The ANOVA revealed a significant main effect of landmark type on recognition performance, *F*(1, 66) = 110.909, *p* < .001, $\eta_{p}^{2}$ = .63, and showed that imagery-incongruent landmarks (*M* = 82.78%, *SEM* = 1.37) could more easily be recognized than imagery-congruent landmarks (*M* = 61.94%, *SEM* = 1.58).

While there was no significant main effect of role adoption task (*F* < 1), there was a significant role adoption task x landmark type interaction, *F*(2, 66) = 5.757, *p* = .005, $\eta_{p}^{2}$ = .15. A post-hoc analysis (Bonferroni corrected) showed that squobbles (*M* = 65.83%, *SEM* = 3.05) performed significantly better than the control condition (*M* = 56.39%, *SEM* = 2.48) when imagery-congruent landmarks were used, *p* = .023. Additionally, rats (*M* = 63.61%, *SEM* = 1.99) were almost on the same level as squobbles. All groups were significantly better than chance level (50%).

**Fig 6:**
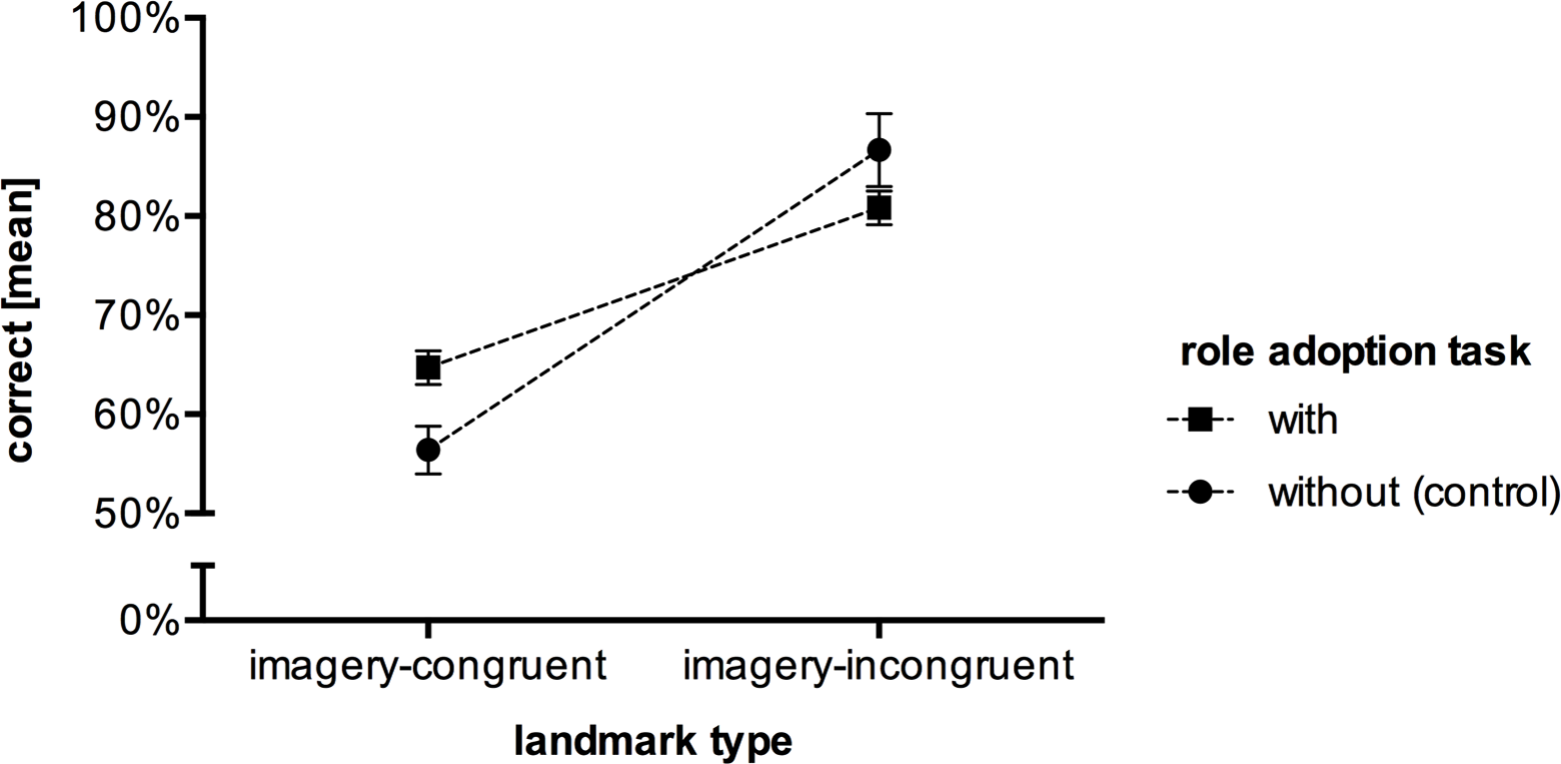
Mean response accuracy of the landmark recognition task in Experiment 2. Please note that pooled data points are depicted for the experimental group, who conducted a role adoption task. The error bars represent the *SEM*.

Afterwards, since there were no differences between ‘rats’ and ‘squobbles’, we pooled the data and compared the performance between those who received a role adoption task and those who did not (same as in Experiment 1). The results (Fig 6) of the 2x2 ANOVA revealed a significant landmark type x role adoption task interaction, *F*(1, 68), = 11.663, *p* = .001, $\eta_{p}^{2}$ = .15. Post-hoc analysis demonstrated that the role adoption task facilitated the recognition of imagery-congruent landmarks (*p* = .006) and hindered the recognition of imagery-incongruent landmarks (*p* = .051).

Lastly, we analyzed the response time data. However, there was only a significant main effect of landmark type, *F*(1, 66) = 12.497, *p* = .001. Subjects in the imagery-congruent landmark condition (*M* = 1.57 s, *SEM* = .09) were significantly slower than subjects in the imagery-incongruent landmark condition (*M* = 1.21 s, *SEM* = .05).

### Discussion

In Experiment 2, we let subjects encode landmark information in a dynamic environment (virtual maze). After that, we tested whether subjects could recognize the landmarks they previously had seen. Our results show that guided mental imagery leads to superior recognition of imagery-congruent landmarks and worse recognition of imagery-incongruent landmarks. These findings are in line with our hypothesis and further strengthen our results of the previous experiment. In fact, it shows that even in a more difficult environment (dark dynamic scene) where people can freely look around, the role adoption task can facilitate or impair landmark recognition performance.

In contrast to Experiment 1, we did not find support for our hypothesis in terms of subject’s response times. Subjects were generally faster in recognizing imagery-incongruent landmarks (i.e. random animal pictures). One simple reason for that might have been that the content of these landmarks were clearer. For example, it might be easier to detect an elephant compared to a valve. Nevertheless, our results demonstrate the interaction effect in object recognition despite these facts.

Assessing recognition performance can be a sensitive tool for investigating the role of landmarks in wayfinding in general. They are valuable because in environments which feature landmarks, associations between movement decisions and visual cues can be learned [17, 33]. The observation that all these differences were visible in the recognition task is in line with other research. For example, even when it comes to different landmark processing modalities (e.g., visual versus acoustic), differences are predominantly visible within recognition performance [32].

In addition to the role adoption task of Experiment 1, we created another one that consisted of a fictitious character. We did so in order to control for prior knowledge. This is also critically discussed by Shapiro [34], who concludes that the use of fictional stimuli does not remove domain knowledge. One method to overcome this gap would be to assess prior knowledge separately as a subject variable. Although the results did not show any significant differences between the two role adoption tasks, we could see this as a limitation of our study.

## General discussion

The current study sought to examine the role of mental imagery within spatial cognition. Based on our theoretical foundation we proposed the *Imagery-Induced Interaction Hypothesis* (3IH). On a general level, this hypothesis states that mental imagery influences the encoding of object information positively or negatively, depending on the semantic congruence of object and imagined concept. We tested this in a series of two experiments. The first experiment consisted of a static scene. The second experiment was conducted in a dynamic scene by using a virtual environment. In both experiments, a role adoption task (by means of guided imagery) was given to subjects of the experimental group. Additionally, we used objects (landmarks) that were either imagery-congruent or imagery-incongruent. Summarized, our results corroborate the 3IH.

How can we explain this effect? In the beginning of this paper we presented the controversy around mental imagery and discussed three different aspects that built our theoretical foundation. First, mental imagery can change how we perceive our environment. For example, priming can facilitate object recognition, at the same time anticipating one object can lead to overlooking another one (mental imagery and perception). Second, mental imagery can change the extent to which objects become important and meaningful to us (mental imagery and semantic salience). Finally, mental imagery can influence to what extent information is being encoded and retrieved later on (mental imagery and memory). Obviously, all these three elements interact with each other and cannot account for our results separately. The reason for introducing this trichotomy was to structure this paper in a manner that corresponds to findings in different research areas. Nevertheless, these findings underline our hypothesis.

One could also think of an analogy in the human visual system. After a while in the dark, rods and even more cones increase their sensitivity (dark adaption) due to visual pigment regeneration [35]. Turning the light on suddenly leads then to the perception of a glare. In the same manner, the role adoption task could have temporarily increased sensitivity for certain stimuli (i.e. imagery-congruent landmarks). Concurrently, imagery-incongruent landmarks may have led to an oversaturation and thus to a worse performance because of their high visual salience (more on that account in the subsection about limitations). Of course, it is difficult to take an analogy from the area of low-level processing but it shows that more research is needed when it comes to the question how mental imagery influences both high- and low-level processing.

Other accounts can be derived from neuroimaging studies. For example, the retrosplenial/parietal-occipital sulcus region plays an important role in integrating path and landmark information [36]. The same region is also known to be important when it comes to mental images and perspective priming [37, 38]. Imagination tasks could therefore interact during the correct integration of path and landmark information. While rough path integration is important, the creation of a cognitive (communicable) map, which is often tested by asking subjects to report survey knowledge, is less so. For example, Foo et al. [39] investigated whether subjects integrate novel routes into a cognitive map or simply rely on landmarks. The authors found that, only when landmarks were perceived as unreliable, subjects fell back on survey knowledge.

We would like to encourage other researchers to further investigate this topic with respect to perceptual and cognitive accounts in order to understand the mechanisms behind such an interaction to the fullest.

### Alternative accounts

Alternative constructs such as *theory of mind*, *self-efficacy*, *or embodiment* cannot explain our findings very well. The term theory of mind stands for our ability to attribute certain mental states to others as well as oneself and is, for example, impaired in children with autism [40]. This ability cannot explain our findings to the fullest. While we agree that subjects should be able to attribute mental states to the adopted role, the role adoption task was accomplished by means of guided imagery. That means that subjects were guided on a (imagined) visual and cognitive level (e.g., “You know these tunnels”).

This leads to the idea that self-efficacy could have played a role. Self-efficacy describes the case where expectations of personal efficacy can predict performance [41]. However, the fact that there was no main effect of role adoption task already shows that mental imagery did not lead to increased performance because of self-efficacy. Of course, we cannot exclude an increase of self-efficacy, however, we then need to find the remaining mechanisms that caused subjects with a role adoption task to perform worse on imagery-incongruent landmarks.

Could one of those mechanisms be related to embodiment, which is the way we experience a certain body as ours? We believe that this is not the case. In the well-known rubber-hand illusion, for example, synchronous visuo-tactile stimulation of an artificial hand (rubber-hand) and one’s own hand leads to tactile sensations referred to the alien limb [42]. Technological progress allowed the extension of this line of research. For example, by the means of virtual reality, it could even be shown that the so-called full-body illusion can lead to physiological changes such as a reduction of skin temperature [43]. In our second experiment, we ensured free head movement of the avatar in the virtual environment. While these techniques increase the level of immersion, it is not a sufficient condition for embodiment [44, 45].

It is possible that all three, theory of mind, self-efficacy and embodiment, are crucial when it comes to the role adoption tasks in spatial cognition. However, there is hardly any research available in the spatial cognition literature and not much is known about the respective contributions to such an effect, especially in the context of wayfinding.

### Limitations and future directions

There is one noteworthy aspect that, at first glance, could be seen as a limitation. Imagery-incongruent landmarks had a higher visual salience (e.g., higher in brightness, contrast, etc.). The reason for that is that subjects rated such landmarks as imagery-congruent that were “sewer-themed” (e.g., dark environment, pipes, valves). This should not be a problem in general as long as they can really focus on the landmark. However, in Experiment 2 they were virtually guided through the sewer system which makes it more difficult to get a grasp of the exact object properties. This can explain why there was a main effect of landmark type only in Experiment 2. Despite this “visual strength” or visual salience of imagery-incongruent landmarks, our results demonstrated that mental imagery can overcome such object properties. Subjects who used mental imagery (role adoption task) were still better in the recognition of imagery-congruent landmarks and worse in imagery-incongruent landmarks compared to subjects who did not conduct a role adoption task. Therefore, we believe that this strengthens our findings even more.

Nevertheless, this being one of the first studies in the field, future studies could link mental imagery more closely to spatial cognition. For example, experiments investigating survey knowledge could shed light on other aspects of spatial cognition that were not in the center of this study. Another aspect of mental imagery can be investigated with respect to mental simulation of routes. For example, one line of research could show that such mental simulations are temporally compressed [46]. The authors suggest that these fast simulations allow us to formulate predictions more efficiently in case of slow movements. One could therefore investigate several topics related to that field including how role adoption tasks influence such simulations. Lastly, future studies could consider dividing subjects in good and poor navigators based on a preliminary control experiment. It could be shown that only a spatial secondary task could impair navigation performance of good navigators. Poor navigators were equally impaired in their navigation performance by spatial and verbal secondary tasks [47]. It is possible that the mental imagery interferes with a subsystem of the working memory. Therefore, it could be helpful to differentiate between different types of navigators. Additionally, from the working memory literature it is known that both loading the phonological loop and the visuo-spatial-sketchpad [48, 49] can lead to interferences in wayfinding performance [50].

Lastly, it may be seen as a limitation that our experiment was conducted in a virtual environment. Given the fact that walking is inherently linked to bodily sensations [i.e., during locomotion; 13], our subjects could not encode information, as it is the case in real life. However, by using a head mounted display we could at least ensure free head movement as well as upper body movement. Nowadays, using virtual environments is seen as the (gold) standard when it comes to research in spatial cognition. This is especially the case because space-related knowledge can be generated in real and virtual environments equally well [51]. The findings of such investigations can also be utilized for application purposes. By differentiating correctly and computing mathematical models accordingly, we might design and place landmarks in an optimal way. This can be used in indoor settings such as nursing homes as well as outdoor settings such as crossroads. We could also implement this knowledge into navigation systems and thus direct their users more efficiently. In regards to structural salience of a landmark, Klippel et al. [19] proposed such a mathematical framework for the ideal position of a landmark at an intersection. This was tested by Röser et al. [21] and refined based on the empirical data [52].

In sum, our study showed that mental imagery could indeed play a role in landmark recognition. Still, a lot of research has to be conducted in order to understand the mechanisms behind such an effect. We suggest that the effect we found in our study is somehow linked to processes involving perceptual and higher cognitive mechanisms as well as working memory. Therefore, we plead for a multi-methodological approach for future research.

## Supporting information

S1 Fig Exemplary landmarks. (PDF)

S1 Text Role adoption tasks. (PDF)

S1 Data files The data of each experiment can be found as csv files in this zip-container. (ZIP)
