# Supplementary material for: A rat in the sewer: How mental imagery interacts with object recognition
Source: PLoS One. 2018 Mar 28;13(3):e0194227. doi: 10.1371/journal.pone.0194227 (PMC5874016; doi:10.1371/journal.pone.0194227)
Supplement: S1 Fig — (PDF) [file pone.0194227.s001.pdf]

S1 Fig - Exemplary landmarks

The landmarks we present here are exemplary landmarks (photographed by the authors), similar to those that were used in our experiments:

Imagery-congruent	***Imagery-incongruent (animals)***
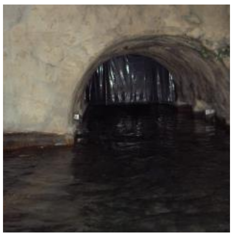	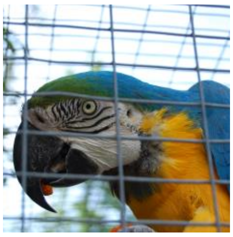
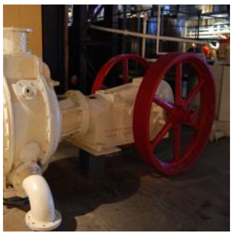	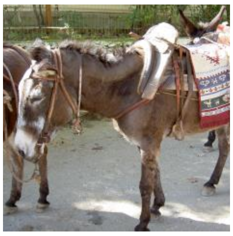
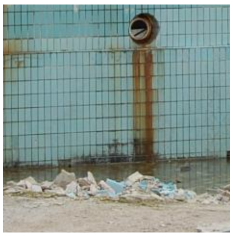	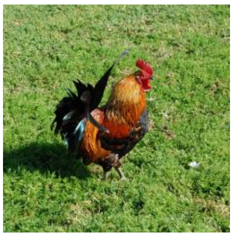
