# Supplementary material for: A rat in the sewer: How mental imagery interacts with object recognition
Source: PLoS One. 2018 Mar 28;13(3):e0194227. doi: 10.1371/journal.pone.0194227 (PMC5874016; doi:10.1371/journal.pone.0194227)
Supplement: S1 Text — (PDF) [file pone.0194227.s002.pdf]

S1 Text - Role adoption tasks

The role adoption tasks were conducted in German. The following role adoption tasks are adapted from German for reviewing purposes and for replication studies by the scientific community.

Rat condition

Sit down and make yourself comfortable. Close your eyes and then breathe deeply. Imagine you find yourself in a sewer system. You are standing on the curb at the side of the tunnel. However, the curb is much bigger than you are used to. You look down to your hands, but, instead of your hands, you realize that there are two tiny paws with fine claws. You look down on yourself. Your body is covered of grey fur. Your feet became clawed paws as well. As you look down, a puddle of water reflects your image: You are a rat. You have a pointy nose with long whiskers and a long and pink tail. Your tiny black button eyes gleam in the dark. Because of your transformation, everything seems bigger than usual. You can smell better: the smell of wet stones…a rat, which is waiting nearby…a rusty can. And, despite the darkness, you know exactly how to walk to find these things. You know these tunnels. They are your home. And you do not have any problems finding your way…

Squobble condition

Sit down and make yourself comfortable. Close your eyes and then breathe deeply. Imagine you find yourself in a sewer system. You are standing on the curb at the side of the tunnel. However, the curb is much bigger than you are used to. You look down to your hands, but, instead of your hands, you realize that there are two tiny paws with fine claws. You look down on yourself. Your body is covered of pink fur. Your feet became clawed paws as well. As you look down, a puddle of water reflects your image: You are a squobble. You have a pointy nose with long whiskers and a long tail. Your tiny black button eyes gleam in the dark. Because of your transformation, everything seems bigger than usual. You can smell better: the smell of wet stones…a rat, which is waiting nearby…a rusty can. And, despite the darkness, you know exactly how to walk to find these things. You know these tunnels. They are your home. And you do not have any problems finding your way…
